# Health facility use by dengue patients in the Klang Valley, Malaysia: a secondary analysis of dengue surveillance data

**DOI:** 10.5365/wpsar.2019.10.1.001

**Published:** 2019-05-21

**Authors:** Yuan Liang Woon, Chiu Wan Ng, Rose Nani Mudin, Zailiza Suli

**Affiliations:** a Clinical Research Centre, Dermatology Block, Hospital Kuala Lumpur, Jalan Pahang, Kuala Lumpur, Malaysia.; b Department of Social and Preventive Medicine, University of Malaya, Jalan Universiti, Kuala Lumpur, Malaysia.; c Sector of Vector-Borne Disease, Disease Control Division, Ministry of Health Malaysia, Putrajaya, Malaysia.

## Abstract

**Background:**

Dengue patients in Malaysia have the choice to seek care from either public or private sector providers. This study aims to analyse the pattern of health facility use among dengue patients to provide input for the ongoing policy discussion regarding public–private integration. The focus of this study is in the Klang Valley, which has a high dengue burden as well as a high number of private facilities.

**Methods:**

This is a cross-sectional study using an available secondary data source – the Malaysian national dengue passive surveillance system, e-Dengue registry. A total of 61 455 serologically confirmed dengue cases from the Klang Valley, registered in year 2014, were included. We retrospectively examined the relationship between demographic factors and the choice of health-care sector by logistic regression.

**Results:**

The median age of the cohort was 26 (interquartile range: 17 to 37) years. More private facilities (54.4%) were used for inpatient care; more public facilities (68.2%) were used for outpatient care. The Chinese and urban populations showed significantly higher use of the private health-care sector with an adjusted odds ratio of 4.8 [95% confidence interval (CI): 4.6–5.1] and 2.3 (95% CI: 2.2–2.4), respectively.

**Conclusion:**

Both public and private health facilities bear significant responsibilities in delivering health-care services to dengue patients. The workload of both sectors should be included in future health policy planning by public agencies.

## Background

Dengue is a fast emerging mosquito-borne viral disease in all World Health Organization (WHO) regions. (*1*) It poses substantial socioeconomic burden to the endemic countries. A recent study reported that both fatal and non-fatal dengue cases contributed to 1.14 million disability-adjusted life-years globally in 2013. (*2*) The disability-adjusted life-years is a measure of disease burden, expressed as the sum of years of potential life lost due to ill health, disability or premature mortality. Furthermore, a prospective study involving three countries in Asia (Cambodia, Malaysia and Thailand) and five countries in the Americas (Brazil, El Salvador, Guatemala, Panama and Venezuela) found that the average cost for an ambulatory dengue case was US$ 514, while the cost for a hospitalized case was US$ 1394. (*3*)

Dengue infection is hyperendemic in Malaysia. Its incidence doubled from 146 per 100 000 population in 2013 to 328 per 100 000 population in 2016. (*4*) It was estimated that Malaysia spent about US$ 175.7 million annually on treatment and dengue prevention activities. (*5*) The Klang Valley, with an estimated population of 7.9 million in the state of Selangor, the Federal Territory of Kuala Lumpur and the Federal Territory of Putrajaya in 2014, has been targeted as the focus for reduction of dengue cases as it has consistently contributed more than half of the national dengue burden. (*4*, *6*–*8*)

The Malaysian health-care system is a mixture of public and private systems; the public and private sectors complement each other in the delivery of health-care services. Public health-care providers serve both rural and urban populations; private health-care providers mainly concentrate in high-density urban areas, especially along the west coast of Peninsular Malaysia. (*9*) The public health-care system is mainly financed through taxation and general revenue collected by the federal government. Additionally, the Employees Provident Fund and Social Security Organization also contributed to health-care funding for the public sector. (*10*) The private sector is mainly funded through private employers, private insurance and out-of-pocket payments. (*10*) To facilitate efficient health policy planning and resource allocation we need to estimate the disease burden in both health-care sectors and understand the factors associated with the pattern of health facility use. In Malaysia, the performance and workload of the private sector were seldom taken into consideration in health policy planning by public agencies. This situation may lead to inefficiencies in the health sector and underutilization of expertise.

Studies related to health facility use in Malaysia are limited. Hence, this study aimed to analyse the pattern of health facility use among dengue patients in the Klang Valley by using the data captured in the national dengue surveillance system, e-Dengue registry.

## Methods

### Study setting and populations

This is a cross-sectional study that is based entirely on an available secondary data source in Malaysia. A secondary data analysis was performed using the data captured in the e-Dengue registry, a national dengue passive surveillance system in Malaysia. All confirmed dengue cases from the Klang Valley registered in the e-Dengue registry from 1 January 2014 to 31 December 2014 were included in the analysis.

We assumed the health facilities that notified cases to the e-Dengue registry were the first medical facilities to be used by dengue patients. For cases with missing information on the name of the medical facility, the full addresses of the health-care facilities were mapped on Google Maps to identify the facilities. Then the facility names were matched with a list of public and private health-care facilities. A list of the Klang Valley public health-care facilities was obtained from the Development Division, Ministry of Health (MoH) Malaysia. The list comprised 18 public hospitals and 89 public clinics. A list of private health-care facilities was obtained from the Medical Practice Division, MoH Malaysia. There were 92 private hospitals and 2205 private clinics in the Klang Valley during the study period.

### The e-Dengue registry and case definition

The e-Dengue registry was established in Malaysia in 2009 under the National Dengue Strategic Plan 2009–2013. (*7*) In Malaysia, it is mandatory for health-care practitioners to notify all clinically suspected or serologically confirmed dengue cases through an online notification system (e-Notice). The diagnosis is verified by district health officers according to the WHO 1997 classification. (*11*) Dengue fever is defined as any case with “acute febrile illness with two or more clinical presentations (myalgia, arthralgia, retro-orbital pain, headache, rash, leukopenia and haemorrhagic manifestations) in additional to supportive serology or occurrence at the same location and time as other confirmed dengue cases. A confirmed dengue case is one that was confirmed through laboratory testing such as dengue virus isolation, virus antigen detection, virus genomic sequence detection and/or a fourfold rise in antibody titer.” (*11*) Only confirmed dengue cases were entered into the e-Dengue registry by the district health officers.

### Statistical analysis

The background characteristics of all dengue patients in the Klang Valley and the pattern of health facility use were described through descriptive analyses. Logistic regression was used to examine the relationship between demographic factors and patients’ choices of health-care sectors. The two-sided statistical significance level was set at *P* < 0.05. Statistical Package for Social Science (version 22.0; SPSS, Chicago, IL, USA) was used for all statistical analyses.

### Ethics approval and consent to participate

This article is research involving secondary data. All cases included in this study were anonymized. The study was registered under the National Medical Research Registry (NMRR-17–544–34899) and approved by the Medical Research Ethics Committee (MREC), MoH Malaysia.

## Results

### Demographic profiles

This study included 61 445 serologically confirmed dengue cases reported within the Klang Valley area in 2014. Patients’ ages ranged from less than one month to 98 years. The age was non-normally distributed, with Kolmogorov–Smirnov normality test showing *P* < 0.001. There were eight cases with missing age. The median age was 26 (interquartile range: 17 to 37) years (**Table 1**). The majority of the cases (77.5%) were adults aged 15–59 years; 18.9% of the cases were children aged below 15 years. Those aged 60 years and above contributed only 3.6% of the total cases. More than half of the cohort were male (57.1%). The majority of the cohort were Malay (54.1%) followed by Malaysian Chinese (25.4%) and Indian (10.3%). In 2014, 8.4% of the registered dengue cases in the Klang Valley were non-Malaysian with the majority from Bangladesh (26.7%) followed by Nepal (23.1%), Indonesia (12.1%), India (6.1%), China (5.3%), Myanmar (5.3%), Pakistan (4.9%) and other countries. Ethnicity information was missing from 778 (1.3%) cases.

**Table 1:** Demographic profiles of confirmed dengue cases in the Klang Valley, 2014

Characteristics	Dengue patients (*n* = 61 445)	
Age in years* (median, IQR)	26	17, 37
**Gender**	***n***	**%**
Male	35 108	57.1
Female	26 337	42.9
**Ethnicity^§^**		
Malay	33 236	54.1
Malaysian Chinese	15 606	25.4
Indian	6354	10.3
Indigenous population	309	0.5
Non-Malaysian	5162	8.4
**Area of residence**		
Urban	52 899	86.1
Rural	8546	13.9

* Eight cases with missing data.

§ 778 cases with missing data.

### Health facility utilization by dengue patients in Klang Valley

**Fig. 1:**
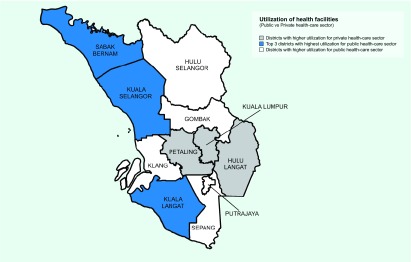
Map of the Klang Valley and the pattern of health facility use

There were 3158 cases with missing information on the health-care sector use. The use of private and public health-care facilities by dengue patients in the Klang Valley was 51% and 49%, respectively. When comparing the use of public and private health-care facilities within the districts, differences of more than 65% were noted for Hulu Selangor district, Kuala Langat district and Sabak Bernam district (**Table 2**). At least 85% of the patients from these districts were using public health-care facilities instead of private health-care facilities. Dengue patients from the Federal Territory of Kuala Lumpur, Petaling district and Hulu Langat district used private health facilities more than public ones. **Fig. 1** shows a map of the Klang Valley.

**Table 2:** Use of health-care facilities of different districts in the Klang Valley

District	Use of health-care facilities, *n*(%)		
	Public sector	Private sector	Difference of public/private*
Gombak	4183 (59.2)	2885 (40.8)	1298 (18.4)
Hulu Langat	5132 (45.5)	6155 (54.5)	−11 023 (−9.0)
Hulu Selangor	1065 (84.8)	191 (15.2)	874 (69.6)
Klang	4493 (73.8)	1597 (26.2)	2896 (47.6)
Kuala Langat	726 (86.0)	118 (14.0)	608 (72.0)
Kuala Selangor	553 (70.4)	232 (29.6)	321 (40.9)
Petaling	9245 (39.5)	14 180 (60.5)	−4,935 (−21.0)
Sabak Bernam	243 (97.2)	7 (2.8)	236 (94.4)
Sepang	328 (64.6)	180 (35.4)	148 (29.2)
Kuala Lumpur	2712 (41.2)	3875 (58.8)	−1,163 (−17.6)
Putrajaya	123 (65.8)	64 (34.2)	59 (31.6)

* Negative values denote more private sector usage than public sector usage.

The utilization rate of hospitals was about five times higher than that of clinics for the whole study population (83% versus 17%). When the utilization of health-care sectors was stratified by type of facility (private versus public), an inverse picture was observed between inpatient and outpatient cases. For hospitalized cases, the use of private facilities (54.4%) was about 10% higher than that of public facilities (45.6%). For cases who presented to outpatient care, the use of private clinics (31.8%) was two times lower than that of public clinics (68.2%).

The association of patients’ demographic profiles and the choice of health-care sectors was tested (**Table 3**). Cases with missing information on health-care sector, age and/or ethnicity (*n* = 3900) were excluded from this analysis. Multivariate analysis showed that females have higher private health care use than males (*P* < 0.001). Compared to the Malay, the Malaysian Chinese were 4.8 times more likely to use private health-care facilities; both the indigenous group and foreigners had lower odds than Malay, with the adjusted odds ratio of 0.2 and 0.5, respectively (*P* < 0.001). The adjusted odds of the urban population choosing private health-care facilities was 2.3 times higher than the rural population (*P* < 0.001).

**Table 3:** Analysis of demographic profile with use of private compared with public health-care facilities

Characteristics	Univariate		Multivariate*	
	Crude OR	95% CI	Adjusted OR	95% CI
**Age group (years)**				
0–9	0.7	(0.5–1.1)	1.0	(0.7–1.6)
10–19	0.5	(0.3–0.7)	0.6	(0.4–0.9)
20–29	0.4	(0.3–0.6)	0.6	(0.4–0.9)
30–39	0.7	(0.5–1.0)	1.0	(0.6–1.5)
40–49	0.9	(0.6–1.3)	1.0	(0.7–1.6)
50–59	0.7	(0.5–1.1)	0.7	(0.5–1.2)
60–69	0.6	(0.4–0.9)	0.6	(0.4–0.9)
70–79	0.6	(0.4–1.0)	0.5	(0.3–0.9)
Above 80	1 (ref.)		1 (ref.)	
**Gender**				
Male	0.8	(0.8–0.8)	0.9	(0.9–0.9)
Female	1 (ref.)		1 (ref.)	
**Ethnicity**				
Malaysian Chinese	4.8	(4.6–5.1)	4.8	(4.6–5.1)
Indian	1.1	(1.0–1.1)	1.1	(1.0–1.1)
Indigenous	0.2	(0.2–0.3)	0.2	(0.2–0.3)
Non-Malaysian	0.6	(0.5–0.6)	0.5	(0.5–0.6)
Malay	1 (ref.)		1 (ref.)	
**Area of residence**				
Urban	2.2	(2.1–2.3)	2.3	(2.2–2.4)
Rural	1 (ref.)		1 (ref.)	

* Analysed with stepwise binary logistic regression, which the model includes age, gender, ethnicity and area of residence; total number of excluded from analysis in view of missing information: 3900 (6.3%).

CI: confidence interval; OR: odds ratio; Ref = reference category used for respective variable in logistic regression.

## Discussion

To the best of our knowledge, this is the first study focused on the pattern of health facility use among dengue patients in Malaysia. We observed similar overall usage of private and public health-care facilities by dengue patients in the Klang Valley. Our finding differs from that reported by Zara AL et al. in which the use of public health-care facilities was two times higher than private health-care facilities among dengue patients in Brazil. (*12*) This difference could be attributed to differences in health-care systems and health-seeking behaviours between Malaysians and Brazilians.

Stratification by the type of health-care facility showed dengue patients in the Klang Valley used more private inpatient care and public outpatient care. The opposite was observed in the National Health Morbidity Survey (NHMS) 2015, which demonstrated a higher utilization of public inpatient care and private outpatient care among the Klang Valley population. (*13*) NHMS is a population survey that targets all types of diseases when collecting information regarding the use of health-care services. (*13*) However, our study involved only confirmed dengue cases. Therefore, the use pattern of health-care services may differ when we focus only on a single medical condition, dengue fever.

Our finding demonstrates that hospital utilization was nearly five times higher than clinic utilization among dengue patients in the Klang Valley. According to a published report, dengue fever is the third and fourth most common discharge diagnosis made in private and public hospitals, respectively, in Malaysia. (*9*) However, Brazil reported the opposite where the majority (81.4%) of the dengue patients used outpatient services. (*12*) The nature of private health insurance and its coverage may influence patients’ health-seeking behaviour. The private insurance policies offered in Malaysia usually cover the cost of hospital admission but not the cost of outpatient care. (*9*) This may have influenced the health-seeking behaviour and caused a propensity for hospital use. The fact that the Klang Valley has the highest percentage of private health insurance coverage (*13*) could possibly explain the higher use of private hospital care.

Similar to the NHMS 2015, we found that private health-care facilities were used most by Malaysian Chinese regardless of the types of facility; the indigenous population had the least private health-care utilization among all. This could be related to the income distribution across the ethnic groups in Malaysia. Malaysian Chinese are generally in a higher income group as compared to other ethnicities, (*14*) and nearly half of Malaysian Chinese were covered by private health insurance.

Malaysia has a mixed public–private provision of health-care services for primary, secondary and tertiary care. Public health-care services are more evenly distributed in urban and rural areas; private facilities tend to be available only in urban areas. (*9*) The population in urban areas may have a higher socioeconomic status and are able to afford private services more than those in the rural population. This may also explain why the urban population has higher use of private health care.

Our study has a few limitations. First, this study only focused on data captured within the Klang Valley, so it might not be suitable to generalize the findings to the whole of Malaysia. Second, as the study was conducted using data from year 2014, the pattern of health facility use might be different now. Third, as the e-Dengue registry only captures confirmed dengue cases, the preference of health-care facilities among the undiagnosed and clinical dengue cases remains unanswered. Fourth, most dengue patients are asymptomatic and might not seek medical attention. Therefore, the high hospital use as observed in this study might be associated with a more severe form of dengue infection. Additionally, we noted the new dengue classification published by WHO in 2009; however, the 1997 WHO dengue classification was the case definition used in the surveillance system at the time of data collection. As this is secondary research, we do not have control over the case definition used by the system. However, a published systemic review concluded no study had formally compared the 1997 WHO dengue classification with the 2009 classification in the area of surveillance and research. (*15*) Therefore, the applicability of either classification in the surveillance system remains unknown. As the e-Dengue registry has limited variables, we were unable to study other factors such as disease severity, economic factors, geographical factors, organizational factors and cultural factors that could potentially play an important role in determining use of health-care facilities. Lastly, as we used the health-care facilities that notified the cases as the proxy for dengue patients’ preferences, it might not reflect the true preference of patients. Nevertheless, this study has a large sample size compared to other studies with similar objectives, and it was the first study in Malaysia to analyse disease-specific health facility use. This study shed light on the big picture of health facility use among dengue patients in the Klang Valley.

## Conclusions

Our results showed both public and private health facilities bear significant responsibilities in delivering health-care services to dengue patients in the Klang Valley. Malaysian Chinese, females and urban populations have higher utilization for private health-care facilities. Future health service and policy planning related to dengue infection should take into account the workload of both public and private sectors.
